# Research on the changes and predictions of the burden of type 2 diabetes mellitus in Pacific Island countries from 1990 to 2019

**DOI:** 10.1371/journal.pone.0293681

**Published:** 2023-12-21

**Authors:** Yan Li, Hao Zhang, Yi Jiang

**Affiliations:** 1 School of Public Health, Chongqing Medical University, Chongqing, China; 2 Research Center for Medical and Social Development, School of Public Health, Chongqing Medical University, Chongqing, China; 3 Department of Endocrine and Breast Surgery, The First Affiliated Hospital of Chongqing Medical University, Chongqing, China; The University of the South Pacific, FIJI

## Abstract

**Aims:**

To assess the burden of type 2 diabetes in Pacific Island countries and predict future trends.

**Methods:**

We analyzed and processed data using R and Excel software, performed Joinpoint 4.7.0 software analysis to investigate changing trends in disease burden, and used an autoregressive integrated moving average model to forecast future trends.

**Results:**

Our study showed that from 1990 to 2019, the burden of type 2 diabetes in Pacific Island countries continues to increase, with the standardized incidence rate showing the most significant growth. Moreover, there were significant differences in the burden of type 2 diabetes between regions. In 2019, American Samoa had the highest standardized incidence rate, while Fiji had the highest standardized death rate and disability-adjusted life year rate. The standardized incidence rate peaked at ages 65–69 years, while the standardized death rate and disability-adjusted life year rate peaked at ages 95 years and 70–74 years respectively. Type 2 diabetes burden was higher among males than females. Based on our forecasting, from 2020 to 2030, the standardized incidence rate is expected to continue to rise, while the standardized death rate and disability-adjusted life year rate will slowly decline.

**Conclusions:**

Our study highlights that the burden of type 2 diabetes in Pacific Island countries has been increasing from 1990 to 2019. Therefore, it is imperative to strengthen disease prevention and control measures in the region.

## 1. Introduction

Diabetes Mellitus has been identified as a significant global public health problem [1], imposing a considerable burden on human life and healthcare systems. Over the period between 1990 and 2019, the mortality rate attributed to diabetes has alarmingly increased by 3% [2]. A remarkable proportion of this phenomenon is attributed to type 2 diabetes mellitus (T2DM), with over 95% of diabetes patients diagnosed with T2DM [3]. T2DM has been found to have adverse effects on health-related quality of life [4] and it is estimated that up to 62%-72% of people with T2DM experience psychological problems, including depression, anxiety, stress, and burnout [5]. The incidence of T2DM has been rising globally over the past two decades, with the Pacific Islands region reporting the highest rates of T2DM [6]. The Pacific Islands region has witnessed a year-on-year increase in the prevalence of T2DM in recent years, which has become one of the primary challenges to the overall health status within the region. The incidence, mortality, and disability rates related to T2DM are the highest for any of the 369 diseases and injuries recorded in the Global Burden of Disease 2019 (GBD2019) database for this region.

Over the past two decades, research on T2DM in Pacific Island countries has mainly focused on influencing factors analysis [7, 8], diabetes prevention strategies [9–13], ethnic differences in the incidence of diabetes in Pacific Island countries [14–17], the relationship between diabetes and other diseases7, and epidemiological research [18, 19]. Some studies specifically highlight the importance of obesity and dietary changes in Pacific Island countries, both of which are thought to be major contributors to the increase in diabetes [7, 20–23]. Additionally, some research has identified challenges faced by Pacific Island countries in responding to diabetes, including insufficient medical resources [7, 24, 25] and public lack of awareness about diabetes [26–28]. Overall, existing research on diabetes in Pacific Island countries has provided valuable information to help us understand and address the current issues related to diabetes. However, there have been few independent studies on the entire type 2 diabetes epidemic in Pacific Island countries, and even fewer researchers have utilized data from the GBD2019 database to examine the burden of T2DM within Pacific Island countries and trends related to burden.

Understanding the trends and projections of the burden of T2DM is crucial for Pacific Island countries to develop effective public health policies. Therefore, the aim of this study is to analyze the burden and trends of T2DM in Pacific Island countries from 1990 to 2019 using data from the GBD 2019 database to project the burden of T2DM in these countries from 2020 to 2030. This will provide valuable insights for the development and optimization of T2DM prevention and treatment strategies in Pacific Island countries, providing scientific evidence and theoretical support for relevant departments and organizations to promote diabetes prevention and treatment efforts in the region. It will also help to alleviate the impact of diabetes on the health of the local population. At the same time, this study can provide key data and insights for improving public health policies and health interventions in Pacific Island countries, contributing to the achievement of sustainable development goals in the region.

## 2. Methods

### 2.1 Data sources

The data source for this study is the Global Burden of Disease 2019 (GBD2019) database, which estimated the global disease burden for 369 diseases or injuries in 204 countries and territories by collecting data from a variety of sources, including censuses, population surveys, vital statistics, disease registries, health service utilization, air pollution detection, satellite imagery, disease notifications, and others. Dismod-MR 2.1, a Bayesian regression modelling tool, was employed together with cause-of-death Pooled models and Spatio-temporal Gaussian regression models to estimate the global disease burden for 369 diseases or injuries in 204 countries and territories. Additionally, the attributable burden of disease for 87 risk factors was computed through systematic analysis [29, 30]. In this study, we have selected T2DM-related morbidity, mortality and DALY data for Pacific Island countries from 1990–2019 using the operational guidelines of the GBD [29]. Additionally, We have analyzed trends in T2DM according to the following 2019 GBD age strata: 10 to14 years, 15 to 19 years, 20 to 24 years, 25 to 29 years, 30 to 34 years, 35 to 39 years, 40 to 44 years, 45 to 49 years, 50 to 54 years, 55 to 59 years, 60 to 64 years, 65 to 69 years, 70 to 74 years, 75 to 79 years, 80 to 84 years, 85 to 89 years, 90 to 94 years, and greater than95 years.

### 2.2 Burden of disease evaluation indicators

#### 2.2.1 Incidence rate

The GBD2019 Integration and Estimation of data across time, sex and age groups using the Bayesian Meta regression tool, DisMod-MR 2.1, to ensure consistency between morbidity and mortality [29].

#### 2.2.2 Death rate

Death rates were calculated for each sex and age group using cause-of-death pooled models and spatial and temporal Gaussian regression models [29].

#### 2.2.3 Disability-adjusted life years(DALY)

DALY is the sum of life years lost due to disease or injury, and is the sum of life years lost due to premature death (YLL) and life years lost due to disability (YLD) [29–31]. YLL can reflect the number of years of life lost due to premature death from disease or injury, based on the number of deaths from disease or injury and the standardized life expectancy for each age group; YLD can reflect the number of years of healthy life lost due to disability from disease or injury, based on the prevalence of the disease or injury and the weighting of the disability.

#### 2.2.4 Average Annual Percentage Change (AAPC)

AAPC is a composite indicator that characterizes the trend of T2DM morbidity or mortality over the entire study duration. After segmenting the entire study period into distinct intervals and calculating the slope for each segment, AAPC is calculated as follows: AAPC = [exp(∑wibi/∑wi)-1] × 100%. In this equation, ‘b’ represents the slope of each segment and ‘w’ represents the length of each segment [32].

### 2.3 Statistical analyses

Data were analyzed and organized using R version 4.2.1 and Excel 2016. The AAPC and its 95% confidence interval (CI) of the standardized incidence, death and DALY rates of T2DM in the Pacific Island countries from 1990 to 2019 were calculated using a pairwise linear regression model in Joinpoint 4.7.0 software of the National Cancer Institute, and their trends were analyzed. AAPC and its 95% CI were used to analyze the trend of change, and the P value corresponding to the linkage point was determined by Monte Carlo permutation testing (α = 0.05 as the test criterion) [33]. AAPC values of >0, <0, and P >0.05 represented an increase in standardized incidence rate, mortality rate, and DALY rate, respectively, over the period of time. The AAPC values of >0, <0 and P >0.05, respectively, represent the increase, decrease and change of standardized morbidity, mortality and DALY rates at that time [34]. The relationships between AAPCs and age-standardized rates, as well as SDIs and AAPCs were calculated using Gaussian process regression and Pearson’s correlation coefficient (r). Statistical significance was defined as P<0.05.

An autoregressive integrated moving average (ARIMA) model was used to predict the age-standardized incidence rate, death rate, and DALY rate of T2DM in Pacific Island countries from 2020 to 2030. ARIMA (p, d, q) models can be used to predict future values based on past values of the variable itself [13]. ARIMA models utilize trends, seasonality, and other features from historical data to predict future trends. The ARIMA model has three components: autoregression (AR), moving average (MA), and differencing (I). Autoregression (AR) builds a predictive model by relating the current value to recent historical values. Moving average (MA) predicts variables at later time steps using prediction errors from earlier time steps. Differencing (I) transforms non-stationary time series into stationary ones to facilitate modeling. The ARIMA model is built using three parameters (p, d, q). Specifically, AR(p) models use p past data points to predict the current value; MA(q) models use the previous q prediction errors to predict the current value; and ARIMA(p, d, q) models combine AR, MA, and differencing operations to analyze and predict a range of time-series data. Four indicators—coefficient of determination (R2), mean absolute percentage error (MAPE), mean absolute error (MAE), and root mean squared error (RMSE)—were used to evaluate the model’s fit in this study.

### 2.4 Definition of T2DM

T2DM was defined as fasting plasma glucose (FPG) ≥ 126 mg/dL (7 mmol/L) or reporting to be on drug or insulin treatment for type 2 diabetes [29]. The determination of T2DM in the GBD study is based on the International Classification of Diseases (ICD-10) codes E11.2, E11.21, E11.22, E11.29 [29].

### 2.5 Definition of SDI

The socio-demographic index (SDI) is a composite measure of a country’s level of development based on three key indicators: average educational attainment, average income, and total fertility rate, with values ranging from 0 to 1. The higher the SDI value, the higher the level of social and economic development of the country [6]. Based on the SDI value, countries can be classified into five levels of development: low (<0.46), low-middle (0.46–0.61), middle (0.61–0.7), middle-high (0.7–0.81), and high (>0.81) [29, 30, 35, 36].

## 3. Results

### 3.1 Change in the Incidence of T2DM

The Age-Standardized Incidence rate of T2DM has been increasing from 1990 to 2019 in Pacific Island countries, with an average annual growth rate of 1.327%. Among all Pacific Island countries, American Samoa had the highest age-standardized incidence rate of T2DM (819.43/100,000), followed by Fiji (797.04/100,000) and Marshall Islands (795.18/100,000), while Guam had the lowest incidence rate (349.01/100,000). Micronesia (Federated States of) was the fastest growth rate, with an average of 2.272% per year, (Table 1).

**Table 1:** The age-standardized incidence rate and temporal trends of T2DM in 1990 and 2019.

Region	1990ASIR (Per100000) NO.(95%CI)	2019ASIR (Per100000) NO.(95%CI)	AAPC NO.(95%CI)
Pacific island countries	345.01(321.57,369.47)	506.06(472.95,542.44)	1.327(1.306,1.349)
American Samoa	541.12(501.3,586.69)	819.43(762.75,882.16)	1.442(1.383,1.5)
Cook Islands	390.07(364.46,417.65)	591.63(556.04,631.45)	1.445(1.428,1.463)
Fiji	529.21(499.14,561.17)	797.04(763.5,835.8)	1.41(1.345,1.474)
Guam	255.86(236.28,278.55)	349.01(320.25,383.34)	1.082(1.045,1.118)
Kiribati	389.63(365.12,417.42)	610.54(568.73,655.39)	1.557(1.497,1.617)
Marshall Islands	499.79(458.93,542.8)	795.18(735.96,858.57)	1.623(1.587,1.659)
Micronesia (Federated States of)	304.31(283.28,327.29)	580.29(540.53,623.06)	2.272(2.154,2.39)
Nauru	318.93(298.08,342.47)	517.47(480.26,557.06)	1.687(1.656,1.718)
Niue	424(389.36,464.62)	703.98(653.63,759.99)	1.767(1.748,1.785)
Northern Mariana Islands	301.2(277.79,328.23)	468.47(432.02,509.81)	1.542(1.487,1.597)
Palau	362.71(338.16,393.1)	607.88(565.49,657.07)	1.801(1.769,1.833)
Papua New Guinea	316.34(292.76,340.35)	463.54(430.14,502.71)	1.334(1.306,1.363)
Samoa	331.84(307.71,358.99)	513.87(473.37,557.77)	1.518(1.478,1.558)
Solomon Islands	283.04(261.62,306.12)	493.91(460.57,528.67)	1.937(1.902,1.971)
Tokelau	311.9(287.53,337.01)	505.3(467.83,553.05)	1.685(1.648,1.723)
Tonga	346.85(323.26,374.26)	501.81(467.88,538.06)	1.285(1.238,1.332)
Tuvalu	311.5(286.61,337.35)	528.08(486.39,573.61)	1.841(1.811,1.871)
Vanuatu	282.19(257.02,308.84)	473.2(435.05,514.52)	1.797(1.752,1.842)

ASIR, age-standardized incidence rate; AAPC, Average annual percentage change; CI, confidence interval.

**Fig 1:**
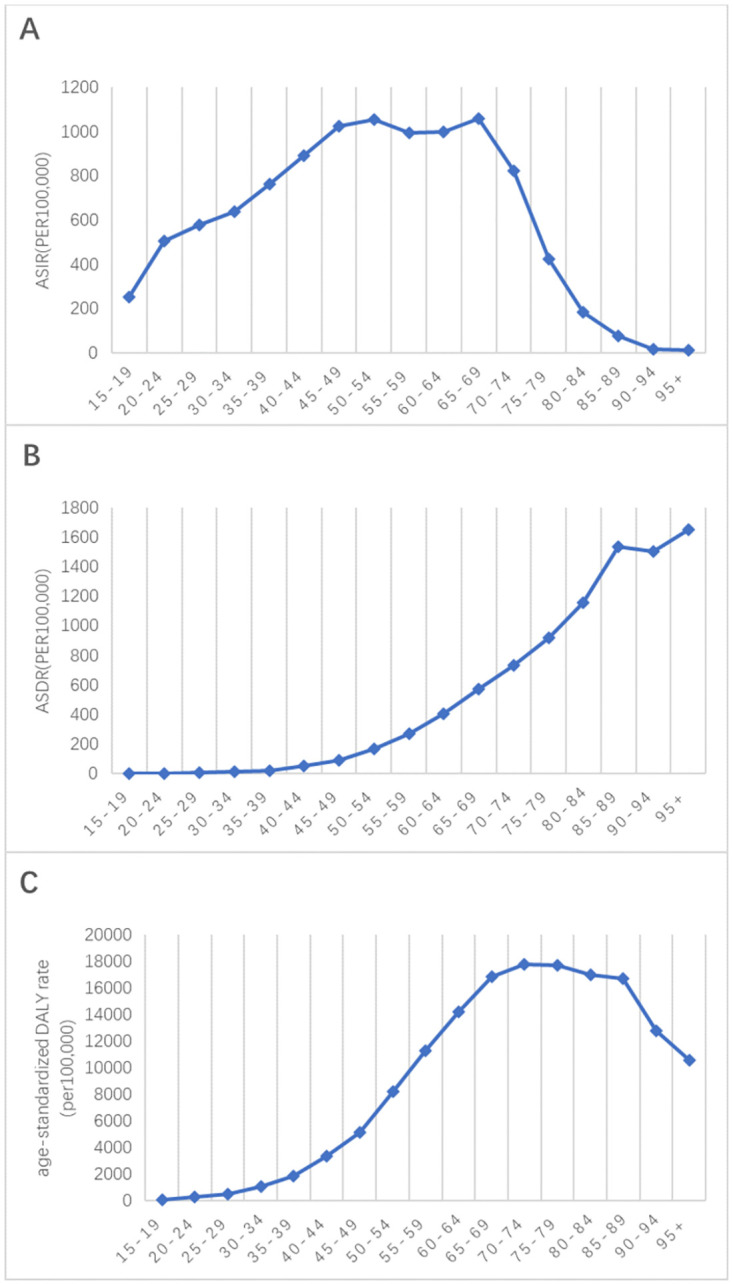
The ration of male to female incidence among different age groups in 2019. ASIR = age standardized incidence rate. ASDR = age standardized death rate. DALY = disability adjusted life-year.

Regarding age groups, the age-standardized incidence rate of T2DM in Pacific Island countries first increased and then decreased. It gradually rises from 15 to 54 years old, slows down from 55 to 64 years old, peaks at 65 to 69 years old, and then rapidly declined (Fig 1A).

**Fig 2:**
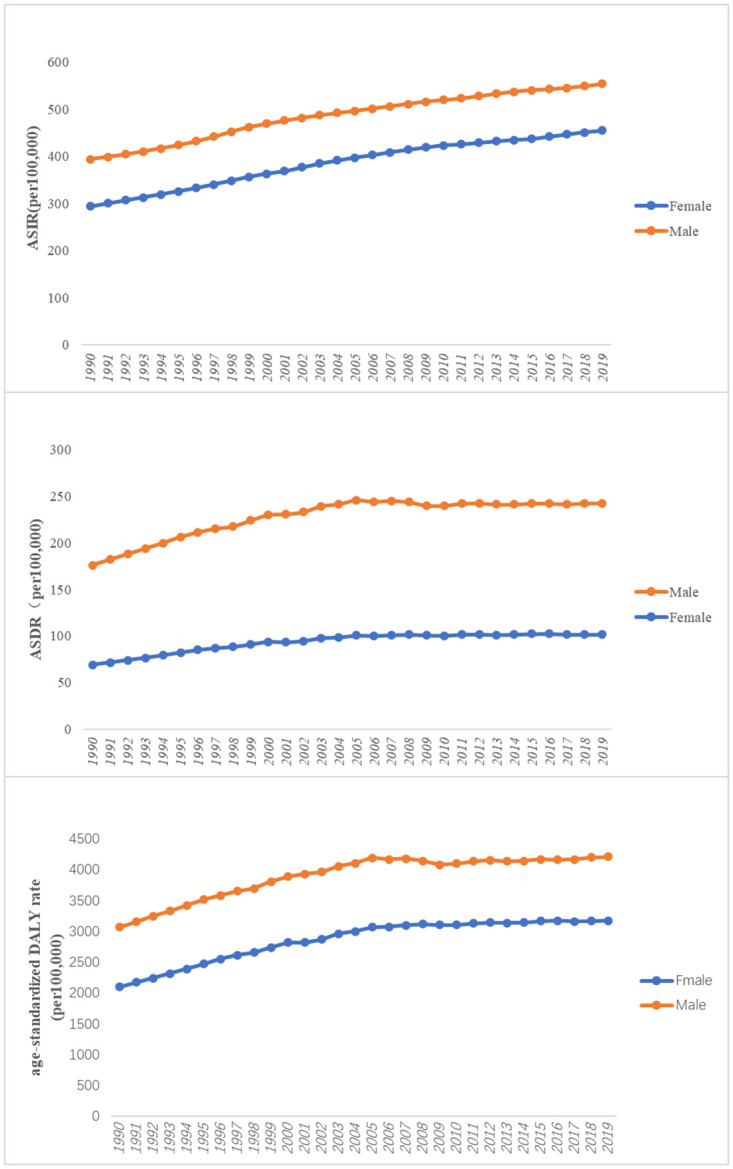
The change trends of age-standardized incidence, death, and DALY rate among different gender from 1990 to 2019.

Regarding gender, the incidence rate of type 2 diabetes in females was lower than that in males for the entire period from 1990 to 2019 (Fig 2).

### 3.2 Change in the death of T2DM

From 1990 to 2019, the age-Standardized Death Rate of T2DM in Pacific Island countries also showed an upward trend, increasing at an average annual rate of 1.105%. The death rate started at 87.72 per 100,000 individuals (95% CI, 73.85 to 107.45) in 1990 and rose to 121.02 per 100,000 individuals (95% CI, 100.23 to 146.5) in 2019. Among all Pacific Island regions, Fiji had the highest age-standardized death rate for T2DM at 257.38/100,000, with a significant increase still observable (AAPC = 1.705). Conversely, Guam had the lowest age-standardized death rate for T2DM and is continuously decreasing, with an average annual decrease of 1.83%. The death rate for the Cook Islands also decreased over the period (AAPC = 0.332). Micronesia (Federated States of) had the fastest growth rate for age-standardized death from T2DM, with an average annual increase of 2.031%. Table 2 presents the data on age-standardized death rates for T2DM in Pacific Island countries.

**Table 2:** The age-standardized death rate and temporal trends of T2DM in 1990 and 2019.

Region	1990ASDR (Per100000) NO.(95%CI)	2019ASDR (Per100000) NO.(95%CI)	AAPC NO.(95%CI)
Pacific island countries	87.72(73.85,107.45)	121.02(100.23,146.5)	1.105(0.984,1.226)
American Samoa	80.53(71.34,94.26)	98.09(84.71,113.59)	0.695(0.488,0.903)
Cook Islands	121.38(105.58,141.63)	110.42(92.78,130.43)	-0.322(-0.523,-0.12)
Fiji	158.1(129.62,203.35)	257.38(210.35,309.25)	1.705(1.162,2.251)
Guam	39.83(33.6,45.49)	23.7(19.89,28.63)	-1.83(-2.302,-1.356)
Kiribati	146.43(120.11,175.84)	203.99(158.28,252.68)	1.151(1.092,1.21)
Marshall Islands	82.83(68.84,99.39)	112.9(86.87,145.94)	1.045(0.825,1.266)
Micronesia (Federated States of)	94.47(75.05,117.11)	169.13(126.79,226.11)	2.031(1.976,2.086)
Nauru	112.79(90.04,144.75)	162.8(126.94,227.02)	1.28(1.162,1.397)
Niue	88.31(72.09,110.56)	122(95.17,162.49)	1.138(1.019,1.256)
Northern Mariana Islands	50.41(43.1,60.4)	58.47(50.02,67.7)	0.391(-0.033,0.818)
Palau	79.06(63.99,98.36)	119.21(92.22,148.24)	1.434(1.343,1.524)
Papua New Guinea	77.11(60.98,97.47)	104.02(82.47,130.12)	1.033(0.931,1.134)
Samoa	71.66(58.02,90.09)	89.18(72.69,111.28)	0.745(0.664,0.825)
Solomon Islands	76.66(56.5,108.4)	133.6(110.61,162.8)	1.902(1.732,2.073)
Tokelau	75.58(60.64,92.84)	96.45(74.96,123.15)	0.831(0.776,0.886)
Tonga	86.25(72.23,100.87)	104.61(83.04,129.12)	0.713(0.474,0.952)
Tuvalu	88.49(72.62,107.63)	115.83(88.37,155.15)	0.944(0.876,1.011)
Vanuatu	43.76(32.14,62.09)	77.53(58.32,103.12)	1.956(1.646,2.268)

ASDR, age-standardized death rate; AAPC, Average annual percentage change; CI, confidence interval.

In terms of age distribution, the age-standardized death rate of T2DM in Pacific Island countries exhibited a linear upward trend, with slow increases in death rates for T2DM before the age of 44 (Fig 1B). A pattern of higher death rates among males was observed when compared with females for T2DM-related deaths (Fig 2).

**Fig 3:**
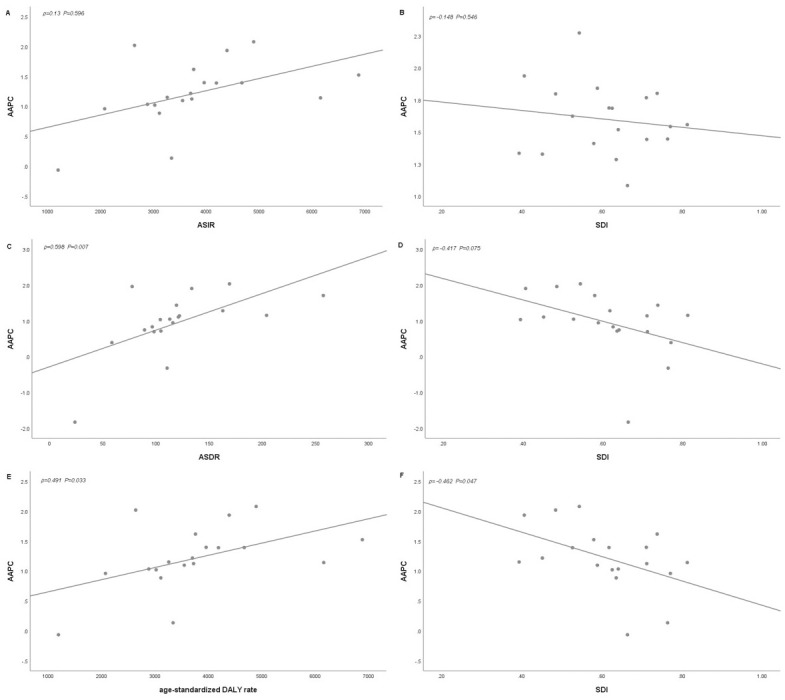
The correlation between AAPC and T2DM age-standardized rates in 1990 and SDI in 2019. The ρ indices Pearson’s correlation coefficient and p values were derived from Pearson’s correlation analysis.

Finally, a positive correlation was found between the average annual percentage change and the age-standardized death rate for T2DM in Pacific Island countries (ρ = 0.598, P = 0.007), as shown in Fig 3C.

### 3.3 Change in the number of DALYs due to T2DM

In line with incidence and death rates, the age-standardized DALY rate for T2DM in Pacific Island countries increased from 1990 to 2019, with an average annual increase of 1.216%. The age-standardized DALY rate for T2DM increased from 2592 per 100,000 (95%CI, 2186.09~3065.54) in 1990 to 3703.43 per 100,000 (95%CI, 3060~4399.34) in 2019. Among all regions in Pacific Island countries, Guam had the lowest age-standardized DALY rate and maintained an average annual decrease of 0.065%. Whether in 1990 or 2019, Fiji and Kiribati ranked first and second in T2DM age-standardization DALY rate (see Table 3).

**Table 3:** The age-standardized DALY rate and temporal trends of T2DM in 1990 and 2019.

Region	1990age-standardized DALY rate (Per100000)NO.(95%CI)	2019age-standardized DALY rate (Per100000)NO.(95%CI)	AAPCNO.(95%CI)
Pacific island countries	2592.69(2186.09,3065.54)	3703.43(3060,4399.34)	1.216(1.138,1.293)
American Samoa	2704.43(2302.73,3174.36)	3725.45(3080.28,4463.18)	1.123(0.981,1.265)
Cook Islands	3221.87(2782.06,3737.09)	3341.81(2821.65,3960.03)	0.135(-0.036,0.305)
Fiji	4357.64(3605.72,5323.4)	6884.3(5667.75,8214.82)	1.523(1.183,1.864)
Guam	1213.52(1032.59,1424.18)	1195.48(951.92,1488.79)	-0.065(-0.303,0.174)
Kiribati	4432.88(3665.62,5242)	6161.41(4876.73,7611.34)	1.141(1.096,1.186)
Marshall Islands	2804.1(2342.64,3381.6)	4191.3(3300.67,5261.92)	1.389(1.205,1.575)
Micronesia (Federated States of)	2718.24(2192.34,3344.85)	4896.31(3682.18,6620.37)	2.078(2.001,2.155)
Nauru	3130.78(2510.26,4072.68)	4673.67(3709.51,6470.25)	1.392(1.289,1.495)
Niue	2665.76(2205.28,3264.06)	3959.98(3166.77,4853.46)	1.395(1.299,1.492)
Northern Mariana Islands	1583.16(1323.41,1873.38)	2076.26(1736.74,2488.53)	0.959(0.657,1.262)
Palau	2367.73(1937.01,2862.02)	3760.83(3008.48,4602.55)	1.617(1.542,1.691)
Papua New Guinea	2331.38(1874.94,2849.2)	3260.52(2613.04,3989.29)	1.15(1.088,1.213)
Samoa	2138.74(1738.43,2629.4)	2887.06(2369.92,3536.54)	1.033(0.963,1.103)
Solomon Islands	2513.15(1927.6,3388.83)	4391.7(3647.6,5319.26)	1.933(1.779,2.087)
Tokelau	2250.69(1812.78,2744.19)	3024.69(2420.85,3766.55)	1.02(0.957,1.082)
Tonga	2433.64(2073.38,2830.94)	3112.61(2552.56,3768.92)	0.885(0.725,1.045)
Tuvalu	2597.9(2159.12,3169.19)	3551.11(2794.1,4647.18)	1.096(1.029,1.163)
Vanuatu	1465.96(1131.08,1965.65)	2641.72(2061.84,3351.17)	2.018(1.731,2.305)

DALY, disability adjusted life-years; AAPC, Average annual percentage change; CI, confidence interval.

In terms of age, the age-standardized DALY rate for T2DM showed an increasing trend with increasing age, with the highest DALY rate observed in the 70 to74 age group (Fig 1C).

There was a positive correlation between the percentage change in the annual average DALY rate for T2DM and the age-standardized DALY rate (ρ = 0.491, P = 0.033), but there was a negative correlation between the percentage change in the annual average DALY rate and SDI (ρ = -0.462, P = 0.047). See Fig 3E and 3F for details.

### 3.4 Forecasting T2DM Burden from 2020 to 2030

#### 3.4.1 Forecast of age-standardized incidence rate

The age-standardized incidence rate for T2DM in Pacific Island countries from 1990 to 2019 was shown to be a non-stationary sequence. After two difference transformations (d = 2) were performed to eliminate the instability of the time series, the autocorrelation function (ACF) and partial autocorrelation function (PAC) indicated that the autocorrelation graph was tailing off, and the partial autocorrelation graph was truncated and censored after the second order. Therefore, we obtained the values of parameters p and q (p = 0, q = 0) for the ARIMA (0,2,0) model, which passed the white noise test (P>0.05). The results of R2 (1.00), MAPE (0.13%), MAE (0.567), and RMSE (0.705) indicate a good fit of the model. Therefore, we choose to use the ARIMA (0,2,0) model to forecast the age-standardized incidence rate of T2DM in Pacific Island countries.

**Fig 4:**
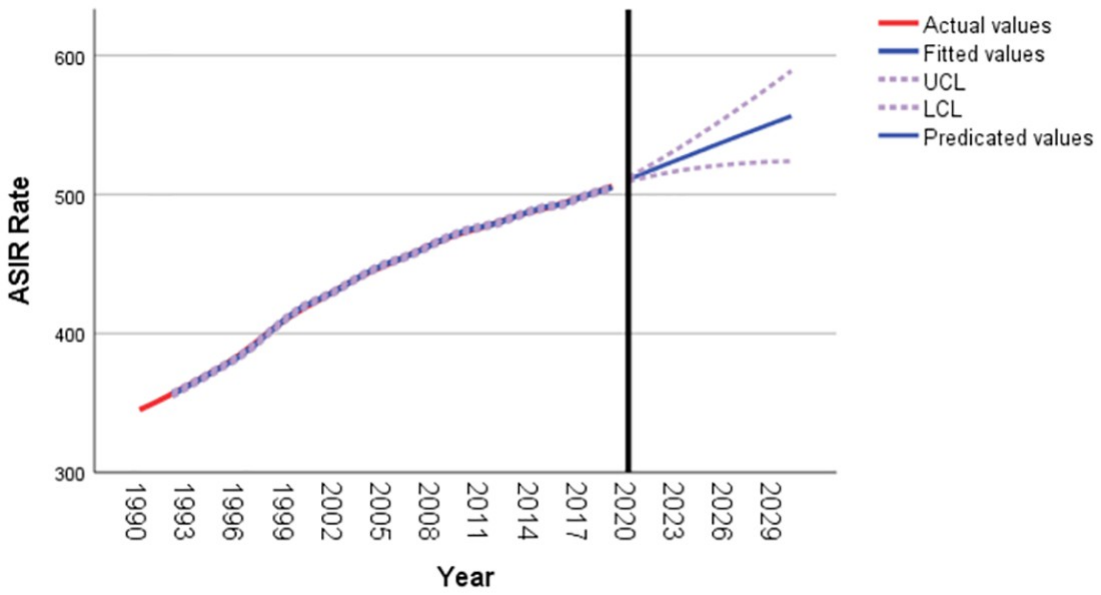
T2DM age-standardized incidence rate forecast for Pacific island countries from 2020 to 2030.

As shown in Fig 4, the age-standardized incidence rate for T2DM in Pacific Island countries is expected to increase overall from 2020 to 2030. It is predicted that the age-standardized incidence rate for T2DM in Pacific Island countries will be 556.36 per 100,000 in 2030, as shown in Table 4.

**Table 4:** Age-standardized incidence rate of T2DM from 2020 to 2030.

Year	Forecast	95%lowerCI	95%upperCI
2020	510.78	509.34	512.23
2021	515.47	512.24	518.70
2022	520.13	514.72	525.54
2023	524.76	516.84	532.68
2024	529.36	518.64	540.09
2025	533.93	520.14	547.73
2026	538.48	521.37	555.59
2027	542.99	522.34	563.65
2028	547.47	523.06	571.89
2029	551.93	523.55	580.31
2030	556.36	523.83	588.89

#### 3.4.2 Forecast of age-standardized death rate

The age-standardized death rate for T2DM in Pacific Island countries from 1990 to 2019 was shown to be a non-stationary sequence. After two difference transformations (d = 2) were performed to eliminate the instability of the time series, the autocorrelation function (ACF) and partial autocorrelation function (PAC) indicated that the autocorrelation graph was tailing off, and the partial autocorrelation graph was truncated and censored after the second order. Therefore, we obtained the values of parameters p and q (p = 0, q = 1) for the ARIMA (0,2,1) model, which passed the white noise test (P>0.05). The results of R2 (0.986), MAPE (0.68%), MAE (0.797), and RMSE (1.031) indicate a good fit of the model. Therefore, we choose to use the ARIMA (0,2,1) model to forecast the age-standardized death rate of T2DM in Pacific Island countries.

**Fig 5:**
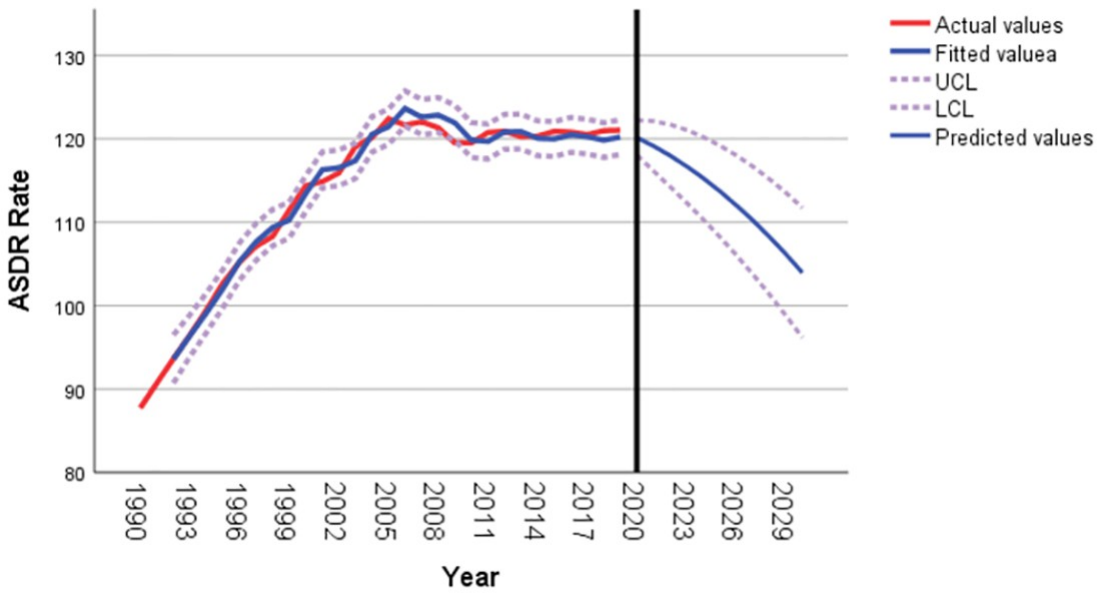
T2DM age-standardized death rate forecast for Pacific island countries from 2020 to 2030.

As shown in Fig 5, unlike the age-standardized incidence rate, the age-standardized death rate for T2DM in Pacific Island countries is expected to decrease overall from 2020 to 2030. It is predicted that the age-standardized death rate for T2DM in Pacific Island countries will be 103.95 per 100,000 in 2030, as shown in Table 5.

**Table 5:** Age-standardized death rate of T2DM from 2020 to 2030.

Year	Forecast	95%lowerCI	95%upperCI
2020	120.14	118.06	122.22
2021	119.13	116.14	122.12
2022	117.99	114.27	121.70
2023	116.70	112.35	121.06
2024	115.29	110.36	120.22
2025	113.73	108.27	119.20
2026	112.05	106.08	118.02
2027	110.23	103.77	116.68
2028	108.27	101.36	115.18
2029	106.18	98.82	113.53
2030	103.95	96.17	111.73

#### 3.4.3 Forecast of age-standardized DALY rate

The age-standardized DALY rate for T2DM in Pacific Island countries from 1990 to 2019 was shown to be a non-stationary sequence. After two difference transformations (d = 2) were performed to eliminate the instability of the time series, the autocorrelation function (ACF) and partial autocorrelation function (PAC) indicated that the autocorrelation graph was tailing off, and the partial autocorrelation graph was truncated and censored after the second order. Therefore, we obtained the values of parameters p and q (p = 0, q = 1) for the ARIMA (0,2,1) model, which passed the white noise test (P>0.05). The results of R2 (0.992), MAPE (0.56%), MAE (19.42), and RMSE (26.382) indicate a good fit of the model. Therefore, we choose to use the ARIMA (0,2,1) model to forecast the age-standardized DALY rate of T2DM in Pacific Island countries.

**Fig 6:**
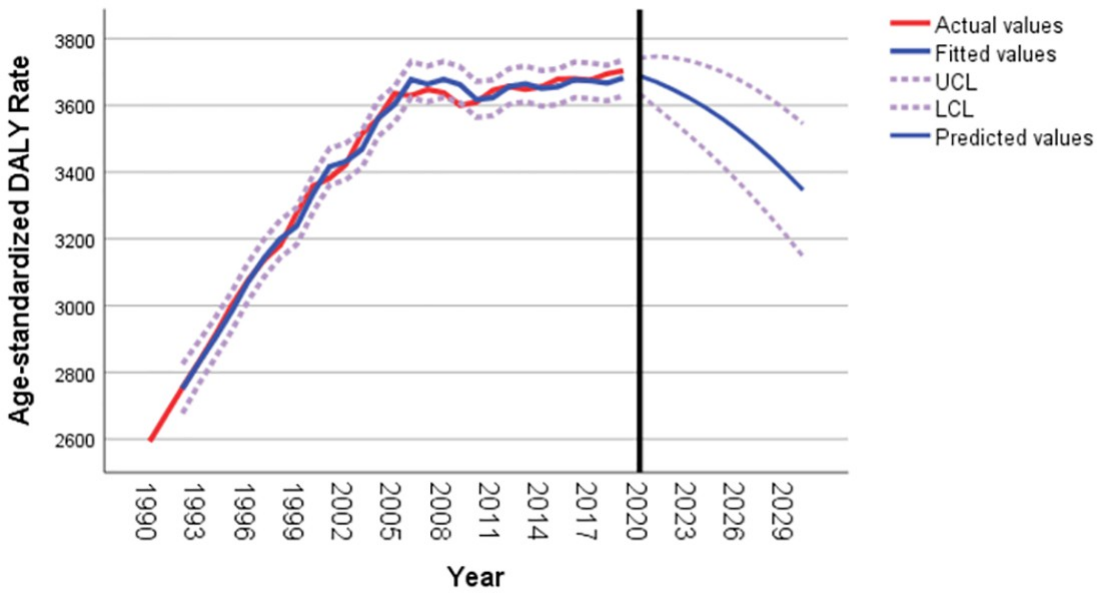
T2DM age standardized DALY rate forecast for Pacific island countries from 2020 to 2030.

As shown in Fig 6, like the age-standardized death rate, the age-standardized DALY rate for T2DM in Pacific Island countries is expected to decrease overall from 2020 to 2030. It is predicted that the age-standardized DALY rate for T2DM in Pacific Island countries will be 3346.2 per 100,000 in 2030, as shown in Table 6.

**Table 6:** Age standardized DALY rate of T2DM from 2020 to 2030.

Year	Forecast	95%lowerCI	95%upperCI
2020	3688.64	3635.47	3741.82
2021	3670.32	3593.88	3746.77
2022	3648.47	3553.41	3743.52
2023	3623.07	3511.76	3734.38
2024	3594.13	3468.04	3720.22
2025	3561.66	3421.84	3701.47
2026	3525.64	3372.90	3678.39
2027	3486.09	3321.05	3651.13
2028	3443.00	3266.19	3619.80
2029	3396.37	3208.24	3584.49
2030	3346.20	3147.14	3545.26

## 4. Discussion

This study aimed to explore the trends of disease burden changes in T2DM among Pacific Island countries and predict future trends over the next 10 years. We have analyzed the disease burden of T2DM in Pacific Island countries based on region, gender, and age, as well as its changing trends over the past 30 years, and predicted the disease burden from 2020 to 2030. The findings of our study have important implications for public health in Pacific Island countries. The results of this study are expected to provide important reference information for healthcare workers, decision-makers, and the general public in the Pacific Island region, promoting the development of T2DM control and prevention efforts in this area.

Firstly, our results show that the burden of T2DM has been increasing in Pacific Island countries, with the standardized incidence rate being the most significant contributor to this rise. According to the research, from 1990 to 2019, the age-standardized incidence rate, death rate, and age-standardized DALY rate of T2DM in Pacific Island countries have all shown an increasing trend. Among these indicators, the age-standardized incidence rate has increased most significantly. The lifestyle in Pacific Island countries is generally simple, with a lack of physical activity and a strong dependence on sugar intake. Numerous studies have confirmed that these factors are one of the main risk factors for the occurrence and progression of T2DM [37, 38]. With the acceleration of economic globalization and industrialization, local traditional dietary structures have gradually been replaced by western-style high-sugar and high-fat diets, which has led to an increased the incidence of T2DM [39, 40]. The increasing incidence of type 2 diabetes is a cause for concern as it can lead to serious comorbidities and increased mortality rates [41]. Therefore, it is essential to strengthen diabetes prevention and control measures in Pacific Island countries to arrest this trend.

Secondly, research has identified significant geographical variations in the burden of T2DM within the Pacific Islands. In 2019, American Samoa had the highest age-standardized incidence rate of T2DM, while Fiji had the highest age-standardized death rate and age-standardized DALY rate of T2DM. In contrast, Guam had the lowest disease burden of T2DM. American Samoa is a place where Polynesian ethnic groups reside, and this ethnic group has a certain degree of genetic susceptibility. In addition, unhealthy dietary habits, lack of physical activity, and socio-economic factors may also increase the risk of diabetes in this region. Fiji, meanwhile, has a high prevalence of chronic diseases like cardiovascular disease and obesity, which often coexist with diabetes and can lead to complications and mortality. Additionally, nutritional deficiencies and limited access to healthcare services in this region may also explain the high diabetes death rate [42, 43]. In contrast, people in Guam maintain traditional dietary habits and focus on outdoor activities, which may help reduce the risk of developing diabetes. Moreover, Guam’s healthcare resources are relatively well-resourced, providing better diabetes prevention and management services. Therefore, different policies should be developed based on the circumstances of different regions to alleviate the burden of T2DM. In American Samoa, targeted genetic research and testing should be conducted to better understand and prevent T2DM [44]. Additionally, genetic susceptibility should be taken into account to develop corresponding prevention measures and guidelines for Polynesian populations. In Fiji, efforts need to be strengthened for chronic disease screening and diagnosis [45] to prevent complications and deaths. At the same time, medical services should be improved to enhance diagnosis and treatment of diabetes. Furthermore, for the entire Pacific Island region, health education should be reinforced to increase awareness and prevention of diabetes. Appropriate policies and regulations should also be established to encourage healthy eating and lifestyle habits to reduce the risk of diabetes.

Thirdly, our study has identified that the age-standardized incidence rate of T2DM peaked at 65–69 years, while the age-standardized death rate increased with age and peaked among those aged 95 years and older. The age-standardized DALY rate reached its zenith in the 70-74bracket. This findings suggests that the prevention and treatment of T2DM should focus on middle-aged and elderly individuals, especially those aged 65–69 years. At the same time, in the treatment and management process, special attention should be paid to the unique needs and health profiles of the elderly. Furthermore, the differences among different age groups must be taken into account in disease control and management efforts to effectively mitigate the severity and risk of the condition.

Fourthly, our study shown that the burden of T2DM was higher among males than females in all Pacific Island countries studied. This finding is consistent with previous studies that have shown a higher risk of T2DM among males compared to females [46, 47]. This may be related to unhealthy dietary patterns and lifestyles, smoking, alcohol consumption, psychological stress, and mental health issues in men. It has been shown that Pacific Island men generally have a higher body mass index(BMI) and waist circumference, which are associated with excessive intake of high-energy, high-fat, and high-sugar foods and lack of exercise [7]. In addition, Pacific Island men engage in more unhealthy behaviors such as smoking and drinking alcohol than women [48]. Therefore, comprehensive measures should be implemented to reduce the disease burden of T2DM in Pacific Island men, including improving lifestyle choices, reducing unhealthy behaviors, and enhancing psychological interventions.

Meanwhile, through data analysis, we found that the age-standardized death rate and age-standardized DALY rate of T2DM in the Pacific region are positively correlated with their respective average annual percentage changes. In addition, there is a negative correlation between the annual average percentage change in age-standardized DALY rate and SDI. This indicates that the death rate and health impact of T2DM in Pacific Island countries are gradually increasing, and this trend is closely related to the level of each country’s economic development. Therefore, when formulating relevant policies, these trends need to be taken into account to strengthen prevention and treatment measures for this disease and reduce its impact on human health.

Lastly, According to the prediction, the age-standardized incidence rate of T2DM in Pacific Island countries is projected to continue to rise from 2020 to 2030, while the age-standardized death rate and age-standardized DALY rate will slowly decline, representing a positive trend. This indicates that the policies and actions taken by Pacific Island countries to control diabetes-related deaths and disabilities are effective. However, it should be noted that the incidence rate remains on the rise, indicating that more efforts are needed to better manage T2DM. Studies have shown that enhancing public awareness education, promoting early diagnosis and treatment, and strengthening government intervention measures have a positive impact on controlling the incidence of T2DM [49, 50]. In Pacific Island countries, people generally have problems with unhealthy dietary structures and lack of exercise; therefore, it is necessary to strengthen public awareness education about healthy diets and lifestyles. In addition, establishing and improving primary medical facilities, enhancing the level of diagnosis and treatment, and carrying out regular screening activities can effectively promote early diagnosis and treatment. The government should adopt more effective policy measures, such as taxing high-sugar foods, providing free or low-cost healthy food options, to reduce people’s consumption of unhealthy food and thus reduce the incidence of diabetes [51].

## 5. Conclusions

In conclusion, our study highlights that the burden of T2DM in Pacific Island countries is increasing and that it is important to strengthen diabetes prevention and control measures in the region. Our study has also forecasted a continuous rise in the standardized incidence rate from 2020 to 2030, while the standardized death rate and disability-adjusted life year rate are expected to slowly decline. This forecasted trend highlights the need for immediate action in Pacific Island countries to prepare for and address the impending diabetes epidemic.

Pacific Island countries should adopt a series of measures to alleviate the burden of T2DM diabetes in the local population. Firstly, health education should be strengthened to raise public awareness and prevent diabetes, promoting healthy lifestyles [52, 53]. Secondly, assistance should be provided to residents to improve their dietary structure, increasing the intake of dietary fiber, whole grains, fruits and vegetables, while reducing the intake of high-sugar, high-fat and high-salt foods [54]. Meanwhile, optimization of medical resource allocation should be implemented to improve the availability and quality of medical care services for diabetes patients. Additionally, strengthening community health services and promoting health insurance can be effective means to provide timely and effective health management and consultation services for diabetic patients and alleviate their economic burden [55]. Finally, international cooperation should be strengthened to introduce advanced medical technologies and management experience, improving the diabetes prevention and control capabilities and levels of Pacific Island countries.

### 5.1 Limitations

This study still has some limitations: the GBD 2019 data are mainly estimates calculated by statistical modelling, which there may be differences with the actual observed data, and it is impossible to avoid the distortion of the results.

## References

[1] International Diabetes Federation, Resources, diabetes L, FAQs, Contact, et al. IDF diabetes atlas [Internet]. [cited 2023 May 31]. https://diabetesatlas.org/

[2] WHO. Diabetes [Internet]. [cited 2023 Apr 12]. https://www.who.int/news-room/fact-sheets/detail/diabetes

[3] Safiri S, Karamzad N, Kaufman JS, Bell AW, Nejadghaderi SA, Sullman MJM, et al. Prevalence, Deaths and Disability-Adjusted-Life-Years (DALYs) Due to Type 2 Diabetes and Its Attributable Risk Factors in 204 Countries and Territories, 1990–2019: Results From the Global Burden of Disease Study 2019. Front Endocrinol (Lausanne). 2022;13:838027. doi: 10.3389/fendo.2022.838027 35282442 PMC8915203

[4] Cannon A, Handelsman Y, Heile M, Shannon M. Burden of Illness in Type 2 Diabetes Mellitus. J Manag Care Spec Pharm. 2018 Sep;24(9-a Suppl):S5–13. doi: 10.18553/jmcp.2018.24.9-a.s5 30156443 PMC10408423

[5] Peyrot M, Rubin RR, Lauritzen T, Snoek FJ, Matthews DR, Skovlund SE. Psychosocial problems and barriers to improved diabetes management: results of the Cross-National Diabetes Attitudes, Wishes and Needs (DAWN) Study. Diabet Med. 2005 Oct;22(10):1379–85. doi: 10.1111/j.1464-5491.2005.01644.x 16176200

[6] Khan MAB, Hashim MJ, King JK, Govender RD, Mustafa H, Al Kaabi J. Epidemiology of Type 2 Diabetes—Global Burden of Disease and Forecasted Trends. J Epidemiol Glob Health. 2020 Mar;10(1):107–11. doi: 10.2991/jegh.k.191028.001 32175717 PMC7310804

[7] Hawley NL, McGarvey ST. Obesity and diabetes in Pacific Islanders: the current burden and the need for urgent action. Curr Diab Rep. 2015 May;15(5):29. doi: 10.1007/s11892-015-0594-5 25809458

[8] Win Tin ST, Kenilorea G, Gadabu E, Tasserei J, Colagiuri R. The prevalence of diabetes complications and associated risk factors in Pacific Islands countries. Diabetes Res Clin Pract. 2014 Jan;103(1):114–8. doi: 10.1016/j.diabres.2013.09.017 24280592

[9] King GL, McNeely MJ, Thorpe LE, Mau MLM, Ko J, Liu LL, et al. Understanding and addressing unique needs of diabetes in Asian Americans, native Hawaiians, and Pacific Islanders. Diabetes Care. 2012 May;35(5):1181–8. doi: 10.2337/dc12-0210 22517939 PMC3329823

[10] Mau MK, Kaholokula JK, West MR, Leake A, Efird JT, Rose C, et al. Translating Diabetes Prevention into Native Hawaiian and Pacific Islander Communities: The PILI ‘Ohana Pilot Project. Prog Community Health Partnersh. 2010; 4(1):7–16. doi: 10.1353/cpr.0.0111 20364073 PMC2852179

[11] Diabetes, mental health, and utilization of mental health professional [Internet]. [cited 2023 Sep 27]. https://www.taylorfrancis.com/chapters/edit/10.4324/9781003152279-4/diabetes-mental-health-utilization-mental-health-professionals-among-native-hawaiian-pacific-islander-adults-angela-fernandez-michael-spencer

[12] Win Tin ST, Laesango N, Gadabu E, Colagiuri R. Comparing metabolic control and complications in type 2 diabetes in two Pacific Islands at baseline and following diabetes care intervention. Journal of Clinical & Translational Endocrinology. 2016 Jun 1;4:32–7. doi: 10.1016/j.jcte.2016.03.001 29159128 PMC5680475

[13] Foliaki S, Pearce N. Prevention and control of diabetes in Pacific people. BMJ. 2003 Aug 21;327(7412):437–9. doi: 10.1136/bmj.327.7412.437 12933734 PMC188499

[14] Hsu WC, Boyko EJ, Fujimoto WY, Kanaya A, Karmally W, Karter A, et al. Pathophysiologic Differences Among Asians, Native Hawaiians, and Other Pacific Islanders and Treatment Implications. Diabetes Care. 2012 May;35(5):1189–98. doi: 10.2337/dc12-0212 22517940 PMC3329855

[15] Bean D, Cundy T, Petrie KJ. Ethnic differences in illness perceptions, self-efficacy and diabetes self-care. Psychology & Health. 2007 Oct 1;22(7):787–811. doi: 10.1080/14768320600976240

[16] Spanakis EK, Golden SH. Race/ethnic difference in diabetes and diabetic complications. Curr Diab Rep. 2013 Dec;13(6):814–23. doi: 10.1007/s11892-013-0421-9 24037313 PMC3830901

[17] Karter AJ, Schillinger D, Adams AS, Moffet HH, Liu J, Adler NE, et al. Elevated rates of diabetes in Pacific Islanders and Asian subgroups: The Diabetes Study of Northern California (DISTANCE). Diabetes Care. 2013 Mar;36(3):574–9. doi: 10.2337/dc12-0722 23069837 PMC3579366

[18] Nanditha A, Ma RCW, Ramachandran A, Snehalatha C, Chan JCN, Chia KS, et al. Diabetes in Asia and the Pacific: Implications for the Global Epidemic. Diabetes Care. 2016 Mar;39(3):472–85. doi: 10.2337/dc15-1536 26908931

[19] Zimmet P. Nauru and mauritius: barometers of a global diabetes epidemic. Hamdan Medical Journal. 2010 Aug;3(2):78. Available from: https://journals.lww.com/hmmj/Abstract/2010/03020/Nauru_and_mauritius__barometers_of_a_global.1.aspx

[20] McLennan AK, Ulijaszek SJ. Obesity emergence in the Pacific islands: why understanding colonial history and social change is important. Public Health Nutr. 2015 Jun;18(8):1499–505. doi: 10.1017/S136898001400175X 25166024 PMC10271720

[21] McElfish PA, Purvis RS, Esquivel MK, Sinclair KA, Townsend C, Hawley NL, et al. Diabetes Disparities and Promising Interventions to Address Diabetes in Native Hawaiian and Pacific Islander Populations. Curr Diab Rep. 2019 Mar 18;19(5):19. doi: 10.1007/s11892-019-1138-1 30887399 PMC7171975

[22] Oceania University of Medicine Samoa, Lameko V. Obesity in Samoa: Culture, History and Dietary Practices. JSS Volume 10 [Internet]. 2020 Oct 1 [cited 2023 Sep 27];10(10):1–15. https://journal.samoanstudies.ws/2020/09/29/obesity-in-samoa-culture-history-and-dietary-practices/

[23] Aldwell K, Caillaud C, Galy O, Frayon S, Allman-Farinelli M. Tackling the Consumption of High Sugar Products among Children and Adolescents in the Pacific Islands: Implications for Future Research. Healthcare (Basel). 2018 Jul 12;6(3):81. doi: 10.3390/healthcare6030081 30002327 PMC6163880

[24] Zimmet PZ, Alberti KGMM. Epidemiology of Diabetes-Status of a Pandemic and Issues Around Metabolic Surgery. Diabetes Care. 2016 Jun;39(6):878–83. doi: 10.2337/dc16-0273 27222545

[25] Cullen R. The Use of ICT in the Health Sector in Pacific Island Countries. In: Cullen R, Hassall G, editors. Achieving Sustainable E-Government in Pacific Island States. Cham: Springer International Publishing; 2017. p. 305–35. (Public Administration and Information Technology). doi: 10.1007/978-3-319-50972-3_11

[26] Braun KL, Ichiho HM, Kuhaulua RL, Aitaoto NT, Tsark JU, Spegal R, et al. Empowerment through community building: Diabetes Today in the Pacific. J Public Health Manag Pract. 2003 Nov;Suppl:S19–25. 14677326

[27] Tin STW, Lee CMY, Colagiuri R. A profile of diabetes in Pacific Island Countries and Territories. Diabetes Res Clin Pract. 2015 Feb;107(2):233–46. doi: 10.1016/j.diabres.2014.10.010 25467624

[28] Russell L. Poverty, climate change and health in pacific island countries. 2011 Apr 1; https://ses.library.usyd.edu.au/handle/2123/9202

[29] GBD 2019 Diseases and Injuries Collaborators. Global burden of 369 diseases and injuries in 204 countries and territories, 1990–2019: a systematic analysis for the Global Burden of Disease Study 2019. Lancet. 2020 Oct 17;396(10258):1204–22. doi: 10.1016/S0140-6736(20)30925-9 33069326 PMC7567026

[30] GBD 2019 Demographics Collaborators. Global age-sex-specific fertility, mortality, healthy life expectancy (HALE), and population estimates in 204 countries and territories, 1950–2019: a comprehensive demographic analysis for the Global Burden of Disease Study 2019. Lancet. 2020 Oct 17;396(10258):1160–203. doi: 10.1016/S0140-6736(20)30977-6 33069325 PMC7566045

[31] GBD 2019 Viewpoint Collaborators. Five insights from the Global Burden of Disease Study 2019. Lancet. 2020 Oct 17;396(10258):1135–59. doi: 10.1016/S0140-6736(20)31404-5 33069324 PMC7116361

[32] Vidal C, Hoffmeister L, Biagini L. [Trend in cervical cancer mortality in Chile: application of joinpoint regression models]. Rev Panam Salud Publica. 2013 Jun;33(6):407–13. 23939365

[33] Geiss LS, Wang J, Cheng YJ, Thompson TJ, Barker L, Li Y, et al. Prevalence and incidence trends for diagnosed diabetes among adults aged 20 to 79 years, United States, 1980–2012. JAMA. 2014 Sep 24;312(12):1218–26. doi: 10.1001/jama.2014.11494 25247518

[34] Armstrong RA. When to use the Bonferroni correction. Ophthalmic Physiol Opt. 2014 Sep;34(5):502–8. doi: 10.1111/opo.12131 24697967

[35] GBD 2019 Risk Factors Collaborators. Global burden of 87 risk factors in 204 countries and territories, 1990–2019: a systematic analysis for the Global Burden of Disease Study 2019. Lancet. 2020 Oct 17;396(10258):1223–49. doi: 10.1016/S0140-6736(20)30752-2 33069327 PMC7566194

[36] GBD 2017 Disease and Injury Incidence and Prevalence Collaborators. Global, regional, and national incidence, prevalence, and years lived with disability for 354 diseases and injuries for 195 countries and territories, 1990–2017: a systematic analysis for the Global Burden of Disease Study 2017. Lancet. 2018 Nov 10;392(10159):1789–858. doi: 10.1016/S0140-6736(18)32279-7 30496104 PMC6227754

[37] Abdul-Ghani MA, DeFronzo RA. Pathogenesis of Insulin Resistance in Skeletal Muscle. J Biomed Biotechnol. 2010;2010:476279. doi: 10.1155/2010/476279 20445742 PMC2860140

[38] Sanghani NB, Parchwani DN, Palandurkar KM, Shah AM, Dhanani JV. Impact of lifestyle modification on glycemic control in patients with type 2 diabetes mellitus. Indian J Endocrinol Metab. 2013 Nov;17(6):1030–9. doi: 10.4103/2230-8210.122618 24381880 PMC3872681

[39] Lin X, Xu Y, Pan X, Xu J, Ding Y, Sun X, et al. Global, regional, and national burden and trend of diabetes in 195 countries and territories: an analysis from 1990 to 2025. Sci Rep. 2020 Sep 8;10(1):14790. doi: 10.1038/s41598-020-71908-9 32901098 PMC7478957

[40] Zhang N, Du SM, Ma GS. Current lifestyle factors that increase risk of T2DM in China. Eur J Clin Nutr. 2017 Jul;71(7):832–8. doi: 10.1038/ejcn.2017.41 28422119

[41] Pradeepa R, Mohan V. Epidemiology of type 2 diabetes in India. Indian J Ophthalmol. 2021 Nov;69(11):2932–8. doi: 10.4103/ijo.IJO_1627_21 34708726 PMC8725109

[42] Lin S, Tukana I, Linhart C, Morrell S, Taylor R, Vatucawaqa P, et al. Diabetes and obesity trends in Fiji over 30 years. Journal of Diabetes. 2016 Jul;8(4):533–43. doi: 10.1111/1753-0407.12326 26201444

[43] Kumar L, Mohammadnezhad M. Health Care Workers’ Perceptions on Factors Affecting Diabetes Self-Management Among Type 2 Diabetes Mellitus Patients in Fiji: A Qualitative Study. Front Public Health.2022;10:779266. doi: 10.3389/fpubh.2022.779266 35444994 PMC9013814

[44] Letourneau LR, Greeley SAW. Congenital Diabetes: Comprehensive Genetic Testing Allows for Improved Diagnosis and Treatment of Diabetes and Other Associated Features. Curr Diab Rep. 2018 Jun 13;18(7):46. doi: 10.1007/s11892-018-1016-2 29896650 PMC6341981

[45] Carmichael J, Fadavi H, Ishibashi F, Shore AC, Tavakoli M. Advances in Screening, Early Diagnosis and Accurate Staging of Diabetic Neuropathy. Front Endocrinol (Lausanne). 2021 May 26;12:671257. doi: 10.3389/fendo.2021.671257 34122344 PMC8188984

[46] Kautzky-Willer A, Leutner M, Harreiter J. Sex differences in type 2 diabetes. Diabetologia. 2023 Jun 1;66(6):986–1002. doi: 10.1007/s00125-023-05891-x 36897358 PMC10163139

[47] Kautzky-Willer A, Abrahamian H, Weitgasser R, Fasching P, Hoppichler F, Lechleitner M. Geschlechtsspezifische Aspekte für die klinische Praxis bei Prädiabetes und Diabetes mellitus. Wien Klin Wochenschr. 2016 Apr 1;128(2):151–8. doi: 10.1007/s00508-016-0957-1 27052235

[48] Baliunas DO, Taylor BJ, Irving H, Roerecke M, Patra J, Mohapatra S, et al. Alcohol as a Risk Factor for Type 2 Diabetes. Diabetes Care. 2009 Nov;32(11):2123–32. 19875607 10.2337/dc09-0227 PMC2768203

[49] Chawla SPS, Kaur S, Bharti A, Garg R, Kaur M, Soin D, et al. Impact of health education on knowledge, attitude, practices and glycemic control in type 2 diabetes mellitus. J Family Med Prim Care. 2019 Jan;8(1):261–8. doi: 10.4103/jfmpc.jfmpc_228_18 30911517 PMC6396605

[50] Dunbar J, Colagiuri S, Reddy P, Vita P, Timoshanko A, Audehm R, et al. Scaling up type 2 diabetes prevention programs: National and State interventions in Australia.

[51] Popkin BM, Hawkes C. Sweetening of the global diet, particularly beverages: patterns, trends, and policy responses. The Lancet Diabetes & Endocrinology. 2016 Feb;4(2):174–86. doi: 10.1016/S2213-8587(15)00419-2 26654575 PMC4733620

[52] Adams RJ. Improving health outcomes with better patient understanding and education. Risk Management and Healthcare Policy. 2010;3:61–72. doi: 10.2147/RMHP.S7500 22312219 PMC3270921

[53] Auld ME, Allen MP, Hampton C, Montes JH, Sherry C, Mickalide AD, et al. Health Literacy and Health Education in Schools: Collaboration for Action. NAM Perspect. 2020;2020. doi: 10.31478/202007b 35291735 PMC8916818

[54] M D. Dietary and Policy Priorities for Cardiovascular Disease, Diabetes, and Obesity: A Comprehensive Review. Circulation. 2016 Dec 1;133(2). doi: 10.1161/CIRCULATIONAHA.115.018585 26746178 PMC4814348

[55] American Diabetes Association Professional Practice Committee. 1. Improving Care and Promoting Health in Populations: Standards of Medical Care in Diabetes-2022. Diabetes Care. 2022 Jan 1;45(Suppl 1):S8–16. doi: 10.2337/dc22-S001 34964872

